# Predictive value of residual active histologic lesions on renal flare in lupus nephritis patients with clinical remission

**DOI:** 10.1093/ckj/sfae350

**Published:** 2024-11-18

**Authors:** Jinhua Hou, Dandan Liang, Songxia Quan, Zhangsuo Liu, Zhihong Liu

**Affiliations:** National Clinical Research Center of Kidney Diseases, Jinling Clinical Medical College of Nanjing Medical University, Nanjing, Jiangsu, China; National Clinical Research Center of Kidney Diseases, Jinling Hospital, Nanjing University School of Medicine, Nanjing, Jiangsu, China; Henan Province Research Center for Kidney Disease, the First Affiliated Hospital of Zhengzhou University, Zhengzhou, Henan, China; Henan Province Research Center for Kidney Disease, the First Affiliated Hospital of Zhengzhou University, Zhengzhou, Henan, China; Traditional Chinese Medicine Integrated Department of Nephrology, the First Affiliated Hospital of Zhengzhou University, Zhengzhou, Henan, China; National Clinical Research Center of Kidney Diseases, Jinling Clinical Medical College of Nanjing Medical University, Nanjing, Jiangsu, China; National Clinical Research Center of Kidney Diseases, Jinling Hospital, Nanjing University School of Medicine, Nanjing, Jiangsu, China

**Keywords:** histologic activity, kidney biopsy, lupus nephritis, renal flare

## Abstract

**Background:**

Renal flare in lupus nephritis (LN) is a crucial contributing factor to poor kidney outcomes. This study aimed at evaluating the predictive value of residual active histologic lesions on renal flare in proliferative LN patients with clinical remission.

**Methods:**

We retrospectively enrolled LN patients with class III/IV ± V (biopsy 1) who had undergone a protocol repeat biopsy (biopsy 2) at 7.3 (IQR: 6.5, 8.4) months after induction therapy with clinical remission and experienced renal flare within 3 years or had been followed up for at least 3 years without renal flare after biopsy 2 with maintenance therapy from two kidney units in China.

**Results:**

A total of 114 eligible patients were included, 28 (24.6%) of whom developed a renal flare. Activity index (AI) at biopsy 2 was significantly associated with LN flare (*P *< .0001). If AI > 1, the OR for LN flare was 23.1 (95%CI, 5.1–103.8, *P *< .001). For patients with partial clinical remission compared with those with complete clinical remission, the OR for LN flare was 3.0 (95%CI: 1.1–8.3, *P *= .029). Multivariate analysis showed that anti-dsDNA positivity, presence of cellular/fibrocellular crescent, and endocapillary hypercellularity at biopsy 2 were independent risk factors for LN flare. When residual active histologic lesions were added to clinical variables, the area under the curve of the prediction model for LN flare significantly increased and the misclassification rate significantly decreased.

**Conclusions:**

Renal flare in LN patients with clinical remission is strongly associated with the residual active histologic lesions.

## Graphical Abstract

**Graphical Abstract:**
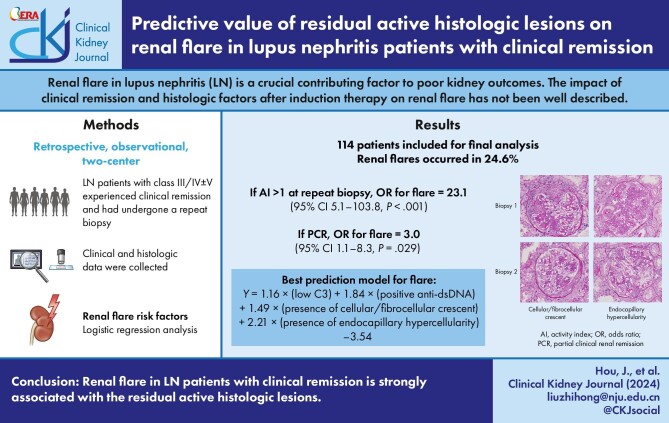

KEY LEARNING POINTS **What was known:**

- The reported flare rate of lupus nephritis (LN) ranged from 20% to 70%, with a flare rate of approximately 20% to 40% within 5 years. Renal flare in LN is a crucial contributing factor to poor kidney outcomes.
- Clinical and histologic factors before induction therapy could not predict LN flare.
- A clinical remission in LN is not equivalent to a histologic remission. The impact of clinical remission and histologic factors after induction therapy on renal flare has not been well described.

**This study adds:**

- The histologic activity index after induction therapy was significantly better than the clinical response in predicting LN flare.
- Patients with complete clinical remission after induction therapy had a better renal flare-free survival than those with partial clinical remission. However, no measurement used to evaluate the clinical remission, such as proteinuria, hematuria, and serum creatinine, was significantly associated with renal flare within 3 years of maintenance therapy.
- The residual active histologic lesions, cellular/fibrocellular crescent and endocapillary hypercellularity, were found to be independent risk factors for LN flare.

**Potential impact:**

- This study underlines the importance of kidney biopsy after induction therapy in the management of LN flare for patients with clinical remission.

## INTRODUCTION

Throughout the entire course of lupus nephritis (LN), patients suffer from renal flare, which is a crucial contributing factor to

poor kidney outcomes. The reported flare rate of LN ranged from 20% to 70%, with a flare rate of ∼20% to 40% within 5 years [1]. The significant differences in LN flare rates reported in clinical trials and cohort studies may be due to the heterogeneity of the study population, and differences in histologic classification, therapeutic regimens, and definition of flare. However, there has been limited research on the clinical and histologic risk factors for LN flare. At present, it is believed that the risk factors for LN flare included African American ethnicity, young onset age, incomplete remission, and serological factors of sustained activity [1]. The few studies that used histologic factors before induction therapy to predict LN flare had inconsistent histologic assessment criteria and conclusions [2–4]. One study reported that patients with World Health Organization type IV were prone to flare [5]. Some studies suggested that the histologic activity and chronicity index before induction therapy could not predict LN flare [4, 6]. Repeated kidney biopsies after induction therapy have confirmed significant changes in histology, which may also be the reason why histologic factors before induction therapy cannot predict renal flare well [7].

Several studies have shown discordance between clinical and histologic findings in patients with LN who have undergone protocol kidney biopsies. A complete clinical remission in LN is not equivalent to a complete histologic remission [7–9]. Otherwise, repeated biopsies in patients with partial clinical remission have shown that some are in histologic remission [7, 10]. Persistent proteinuria may be from past injury and scarring. Thus, we hypothesized that LN patients with clinical remission after induction therapy have a higher likelihood of relapse in the future if they had high histologic AI or accompanied with certain residual active histologic lesions. Therefore, in the present study, we investigated whether histologic findings in LN patients with clinical remission after induction therapy is predictive of LN flare.

## MATERIALS AND METHODS

### Study design and patient selection

This retrospective study design was completed according to the Strengthening the Reporting of Observational Studies in Epidemiology (STROBE) guidelines [11]. The study sample was derived from two kidney units in China, Nanjing Glomerulonephritis Registry at the National Clinical Research Center of Kidney Diseases, Jinling Hospital, and Renal Biopsy Registry System at the First Affiliated Hospital of Zhengzhou University, between January 1999 and December 2013. All patients who fulfilled the 1997 American College of Rheumatology criteria for systemic lupus erythematosus (SLE) and had a biopsy-proven LN, were reclassified according to the 2003 International Society of Nephrology/Renal Pathology Society (ISN/RPS) classification. Patients were included if they (i) had biopsy-proven (biopsy 1) class III/IV ± V LN before induction therapy; (ii) had undergone a repeat biopsy (biopsy 2) while with clinical remission (as defined below) after induction therapy; and (iii) experienced renal flare within 3 years or had been followed up for at least 3 years without renal flare after biopsy 2. Many guidelines recommend immunosuppressive therapy for at least 3 years, during which time discontinuation of immunosuppressant may lead to disease flare, so the time cutoff was chosen to be 3 years and patients who discontinued immunosuppressant within those 3 years were excluded. Patients with missing clinical, pathological, or follow-up data were excluded, as well as those with concomitant kidney diseases.

As this study included patients who underwent renal biopsy with clinical remission, we reviewed the written informed consent of renal biopsy, which was obtained from all patients for the first and second renal biopsies during clinical practice. This study was in compliance with the Declaration of Helsinki and approved by the Ethics Committee of Jinling Hospital (approval number 2019NZKYKS-005-01).

### Renal pathology evaluation

The first and second renal biopsies were re-evaluated by the same nephropathologist (D.L.) without knowing the patient's clinical data, and classified according to the 2003 ISN/RPS classification system [12]. Renal activity and chronic damage were scored using the National Institutes of Health activity index (AI) and chronicity index, respectively [13].

### Collection of clinical and histologic data

Data were collected, including demographic characteristics (gender, age), SLE duration, history of renal flare, history of hypertension, use of angiotensin converting enzyme inhibitors/angiotensin receptor blockers (ACEI/ARB), clinical and pathological features at the first and second renal biopsies, and induction and maintenance treatment regimens. The time of each visit point, as well as laboratory indicators such as urine protein quantification, urine red blood cell levels, serum albumin, and serum creatinine, as well as treatment plans, were collected.

### Definition of remission and flare

Complete clinical renal remission (CCR) was defined as 24-h proteinuria <0.4 g, the absence of active urine sediments, serum albumin >35 g/L and normal or ≤15% increase in serum creatinine (SCr) from baseline. Partial clinical renal remission (PCR) was defined as a decrease in proteinuria by 50% and to between 0.4 and 3.5 g/day, serum albumin >30 g/L and normal or ≤25% increase in SCr from baseline. Patients who met CCR or PCR criteria were considered to be in overall clinical renal remission (OCR). Patients who did not meet CCR or PCR criteria were considered no clinical response [14]. Renal flare was defined by the presence of any one of the following: (i) proteinuric flare: defined as persistent proteinuria ≥1.0 g/day after CCR or an increase of ≥2.0 g/day after PCR and (ii) nephritic flare: an increase in SCr defined as a ≥50% increase in SCr compared with the normal level at OCR or a 30% increase in SCr compared with an abnormal level at OCR [15]. The level of hematuria varied significantly among patients. To prevent the exclusion of LN flares that may not align with the standard definition of renal flares, an arbitrary level of hematuria was not included in the definition of a renal flare [16].

### Statistical analyses

Continuous variables were expressed as medians (IQR) (the data were tested for normality and showed non-normal distribution), and categorical variables were expressed as counts (percentages). Continuous variables between two groups were analyzed using the Mann–Whitney *U*-test (independent samples) or the Wilcoxon signed-rank test (related samples). Fisher's exact test was used for categorical variables. The Spearman correlation was used to test associations. Patients were divided into flare and no-flare groups for comparison. To reduce the number of candidate variables, we excluded lesions with low prevalence (occurring in ≤5 patients), when performing logistic regression analysis. Only one case had cellular crescent at biopsy 2, but there was a statistical difference between the two groups. After discussion with clinical and pathological experts, the fibrocellular crescent was merged with it. Fibrinoid necrosis and karyorrhexis were also combined for analysis. Variables with *P *< .1 in univariate logistic regression analysis were subjected to multivariable logistic stepwise regression (likelihood ratio). Receiver-operating characteristic (ROC) curves and misclassification rates were used to determine the ability of different models to predict renal flare. The cutoff value *C* for predicting LN flare was chosen to maximize the sum of sensitivity and specificity. All other *P* values were two-tailed and considered significant at *P* < .05. All analyses were performed using SPSS v.19.0 (Chicago, IL, USA) and SAS (v.9.1; SAS Institute Inc.).

## RESULTS

Between January 1999 and December 2013, 712 LN patients who underwent repeated kidney biopsies were selected from the Registry Systems. Of all, 121 patients with proliferative LN who achieved complete or partial clinical remission after induction therapy had undergone a per-protocol repeat biopsy. Among them, six patients who did not experience renal flare (four cases with CCR, two cases with PCR) were followed up for <3 years after biopsy 2 (from biopsy 2 to the end of follow-up, the times were 9.7, 12.5, 16.4, 26.2, 30.3, and 33.8 months, respectively). One patient with PCR experienced renal flare after discontinuing immunosuppressive therapy at 2 months after biopsy 2. Excluding these seven patients, a total of 114 patients were included in the analysis. The median time between biopsies 1 and 2 was 7.3 months (IQR: 6.5, 8.4). Most of the patients (78.1%) were biopsied between months 6 and 9 post-biopsy 1. Renal flares occurred in 28 (24.6%) patients during maintenance therapy within a 3-year follow-up period, including 26 with proteinuric flare and 2 with nephritic flare. The median time from biopsy 2 to renal flare was 17.8 months (IQR: 7.9, 24.4). According to whether there was a renal flare within 3 years of maintenance therapy, patients were divided into a flare group (28 cases) and a no-flare group (86 cases). There were no statistically significant differences in the comparison of demographic, clinical, and histologic characteristics between the two groups of patients at biopsy 1 before induction therapy (Table 1).

**Table 1:** Clinical and histologic characteristics at biopsy 1.

Variable	Total (*n* = 114)	Flare group (*n* = 28)	No flare group (*n* = 86)	*P* value^a^
Age (yr)	26.5 (19.6, 34.2)	25.0 (19.7, 35.5)	26.7 (19.5, 34.2)	.887
Female, *n* (%)	98 (86.0)	24 (85.7)	74 (86.0)	1
Duration of SLE (mo)	10.1 (2.1, 41.4)	13.5 (1.8, 49.6)	8.3 (2.3, 40.0)	.703
With prior history of renal flare, *n* (%)	40 (35.1)	11 (39.3)	29 (33.7)	.651
Proteinuria (g/d)	3.04 (2.15, 5.28)	3.90 (2.34, 4.85)	2.83 (2.11, 5.42)	.409
Hematuria (×10^4^/ml)	77 (21, 271)	106 (34, 285)	75 (18, 271)	.345
Serum creatinine (mg/dl)	0.82 (0.61, 1.08)	0.87 (0.60, 1.28)	0.76 (0.61, 1.03)	.280
eGFR (ml/min/1.73 m^2^)	102.0 (72.6, 128.6)	90.4 (58.7, 129.1)	103.6 (76.2, 127.5)	.269
C3 (g/l)	0.366 (0.259, 0.467)	0.315 (0.230, 0.436)	0.369 (0.268, 0.481)	.169
C4 (g/l)	0.088 (0.046, 0.130)	0.078 (0.038, 0.131)	0.093 (0.047, 0.129)	.710
Low C3, *n* (%)	95 (83.3)	26 (92.9)	69 (80.2)	.151
Low C4, *n* (%)	68 (60.2)	18 (64.3)	50 (58.8)	.662
Anti-dsDNA-positive, *n* (%)	79 (69.3)	21 (75.0)	58 (67.4)	.491
SLEDAI-2K	15 (12, 18)	15.5 (13, 18)	14.5 (12, 18)	.492
Activity index	10 (7, 13)	11 (8.3, 13)	9 (6, 13)	.192
Chronicity index	1 (0, 2)	1 (0, 3)	1 (0, 2)	.560
ISN/RPS class				.208
III, *n* (%)	4 (3.5)	0	4 (4.7)	
III + V, *n* (%)	20 (17.5)	3 (10.7)	17 (19.8)	
IV, *n* (%)	50 (43.9)	17 (60.7)	33 (38.4)	
IV + V, *n* (%)	40 (35.1)	8 (28.6)	32 (37.2)	

a For comparison between the flare and no flare groups, a Mann–Whitney *U*-test was used for continuous variables, and Fisher's exact test was used for categorical variables. The data are expressed in median (IQR) or number of cases (%). DAI, disease AI.

The clinical and histologic characteristics at biopsy 2 are summarized in Table 2. Overall, 13.3% (6/45) of patients with CCR experienced renal flare, while 31.9% (22/69) with PCR experienced renal flare. The flare rate of patients with CCR was significantly lower than those with PCR (*P *= .027). The proteinuria in the flare group at biopsy 2 was higher (*P *= .007), while there was no statistically significant difference in hematuria (*P *= .626). The SLEDAI-2 K score in the flare group was significantly higher than that in the no-flare group (*P *< .001). The rates of low C3 (complement component 3), low C4 (complement component 4) and anti-double-stranded DNA (anti-dsDNA) positivity were significantly higher in the flare group. There was no correlation between low C3 and C4 levels, as well as positive rates of anti-dsDNA, and the presence or absence of histologic activity at biopsy 2 (AI = 0 vs AI > 1). Proteinuria at biopsy 2 was not strongly correlated with the AI (Spearman *r* = 0.188, *P *= .045).

**Table 2:** Clinical and histologic characteristics at biopsy 2 after induction therapy.

Variable	Total (*n* = 114)	Flare group (*n* = 28)	No flare group (*n* = 86)	*P* value
Time to OCR (mo)	3.8 (2.5, 6.4)	3.8 (1.9, 6.4)	3.8 (2.5, 6.3)	.567
CCR/PCR, *n*/*n*	45/69	6/22	39/47	.027
SLEDAI-2K	6 (2, 9)	8 (5, 12)	4 (2, 8)	<.001
Proteinuria (g/d)	0.32 (0.21, 0.69)	0.57 (0.31, 0.83)	0.28 (0.20, 0.59)	.007
Hematuria (×10^4^/ml)	5 (1, 32)	8.5 (1, 34)	5 (1, 31)	.626
Serum creatinine (mg/dl)	0.67 (0.57, 0.83)	0.74 (0.58, 0.93)	0.67 (0.57, 0.81)	.405
eGFR (ml/min/1.73 m^2^)	118.7 (100.9, 131.0)	110.9 (97.4, 128.0)	120.9 (100.9, 131.5)	.453
Low C3, *n* (%)	23 (20.2)	11 (39.3)	12 (14.0)	.006
Low C4, *n* (%)	19 (16.7)	9 (32.1)	10 (11.6)	.018
Anti-dsDNA-positive, *n* (%)	28 (24.6)	13 (46.4)	15 (17.4)	.004
AI	1.5 (0.8, 4)	4 (3, 6)	1 (0, 3)	<.001
Chronicity index	3 (2, 4)	3 (2, 4)	3 (2, 3.3)	.094
ISN/RPS class				.008
II, *n* (%)	13 (11.4)	1 (3.6)	12 (14.0)	
III, *n* (%)	42 (36.8)	13 (46.4)	29 (33.7)	
III + V, *n* (%)	33 (28.9)	6 (21.4)	27 (31.4)	
IV, *n* (%)	4 (3.5)	3 (10.7)	1 (1.2)	
IV + V, *n* (%)	4 (3.5)	3 (10.7)	1 (1.2)	
V, *n* (%)	18 (15.8)	2 (7.1)	16 (18.6)	
Cellular crescent, *n* (%)^a^	11 (9.6)	10 (35.7)	1 (1.2)	<.001
Fibrocellular crescent, *n* (%)^a^	33 (28.9)	15 (53.6)	18 (20.9)	.001
Cellular/fibrocellular crescent, *n* (%)^a^	37 (32.5)	18 (64.3)	19 (22.1)	<.001
Endocapillary hypercellularity, *n* (%)^a^	36 (31.6)	19 (67.9)	17 (19.8)	<.001
Leucocyte exudation, *n* (%)^a^	3 (2.6)	2 (7.1)	1 (1.2)	.149
Fibrinoid necrosis/karyorrhexis, *n* (%)^a^	13 (11.4)	8 (28.6)	5 (5.8)	.003
Hyaline deposits, *n* (%)^a^	9 (7.9)	4 (14.3)	5 (5.8)	.220
Microthrombus, *n* (%)^a^	2 (1.8)	1 (3.6)	1 (1.2)	.433
Subendothelial deposits, *n* (%)^a^	24 (21.1)	11 (39.3)	13 (15.1)	.014
Subepithelial deposits, *n* (%)^a^	90 (78.9)	22 (78.6)	68 (79.1)	1
Acute tubular injury, *n* (%)^a^	17 (14.9)	8 (28.6)	9 (10.5)	.031
Interstitial inflammation, *n* (%)^a^	70 (61.4)	21 (75.0)	49 (57.0)	.118
Tubulitis, *n* (%)^a^	9 (7.9)	4 (14.3)	5 (5.8)	.220
Hyalinosis, *n* (%)^a^	24 (21.1)	5 (17.9)	19 (22.1)	.792
Noninflammatory necrotizing vasculopathy, *n* (%)^a^	1 (0.9)	1 (3.6)	0	.246
True renal vasculitis, *n* (%)^a^	0	0	0	
TMA, *n* (%)^a^	1 (0.9)	1 (3.6)	0	.246
Arterial intimal fibrosis, *n* (%)^a^	41 (36.0)	9 (32.1)	32 (37.2)	.659

^a^Number (percentage) of patients at biopsy 2.

DAI, disease AI; TMA, thrombotic microangiopathy.

The AI in the flare group at biopsy 2 was significantly higher than that of the no-flare group (*P *< .001). The distribution of AI in two groups at biopsy 2 is provided in Supplementary Figure S1. Among 114 patients, 28 (24.6%) achieved complete histologic remission (AI = 0). At biopsy 2, 64.4% (29/45) of patients with CCR had persistent histologic activity. The histologic components of active, chronic, and vascular lesions at biopsy 2 were examined in each patient. Cellular/fibrocellular crescent, endocapillary hypercellularity, fibrinoid necrosis/karyorrhexis, subendothelial deposits, and acute tubular injury in the flare group were significantly higher than those in the no-flare group. However, there were no statistically significant differences in the chronicity index and chronic lesions, including global sclerosis, segmental sclerosis, fibrous crescent, tubular atrophy, and interstitial fibrosis, between the two groups. Vascular lesions including hyalinosis, noninflammatory necrotizing vasculopathy, true renal vasculitis, thrombotic microangiopathy, and arterial intimal fibrosis were no statistically significant differences between the two groups.

There were no differences in the induction and maintenance treatment regimens between the flare and no-flare groups (*P *= .115 and *P *= .346, respectively). (Supplementary Table S1).

### Risk factors and prediction models of LN flare

Logistic regression analysis showed a significant association (*P *< .001) between AI at biopsy 2 and the LN flare within 3 years during maintenance immunosuppression. If the AI > 0, the odds ratio (OR) for LN flare was 12.4 [95% confidence interval (CI): 1.6–95.7, *P *= .016], with a sensitivity of 96.4% and specificity of 31.4% for predicting renal flare. The area under the curve (AUC) was 0.64, and the misclassification rate was 24.6%. If the AI > 1, the OR for LN flare was 23.1 (95%CI: 5.1–103.8, *P *< .001), the sensitivity for predicting renal flare was 92.9%, the specificity was 64.0%, the AUC was 0.78, and the misclassification rate was 24.6%. For patients who achieved PCR compared to CCR, the OR for LN flare was 3.0 (95%CI: 1.1–8.3, *P *= .029) and the sensitivity, specificity, AUC, and misclassification rate of predicting renal flare were 78.6%, 45.3%, 0.62, and 24.6%, respectively.

Univariate predictors are provided in Supplementary Table S2. We found that SLEDAI-2 K, proteinuria, low C3 and C4 levels, and positive anti-dsDNA at biopsy 2 were potential predictors for LN flare. The AI at biopsy 2, as well as the presence of cellular/fibrocellular crescent, endocapillary hypercellularity, fibrinoid necrosis/karyorrhexis, subendothelial deposits, and acute tubular injury, were potential predictors in histologic lesions for LN flare. However, the chronicity index (OR: 1.304, 95% CI: 0.926–1.836, *P *= .129) and chronic lesions at biopsy 2, including global sclerosis, segmental sclerosis, fibrous crescent, tubular atrophy, and interstitial fibrosis, were not significantly associated with the odds of an LN flare.

**Figure 1:**
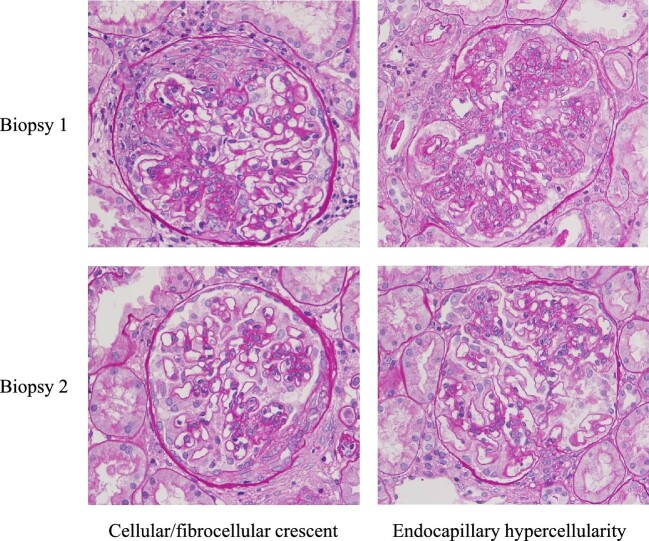
The residual active histologic lesions after induction therapy in lupus nephritis patients with clinical remission were independent risk factors for LN flare. The photographs came from two different patients with cellular/fibrocellular crescent and endocapillary hypercellularity, respectively (periodic acid-Schiff, ×400).

Using the significant predictors identified through univariate analysis (*P *< .1), a multivariable logistic stepwise regression analysis was conducted to determine the predictive factors for LN flare within 3 years of maintenance immunosuppression (Table 3, Fig. 1), and multivariable logistic regression models were developed to predict future LN flare (Table 4). When histologic variables were added to clinical variables, the area under the ROC curve of the prediction model significantly increased and the misclassification rate significantly decreased. And specific clinical variables combined with specific histologic variables predicted LN flare relatively better than scoring indicators such as SLEDAI-2 K and AI. If *Y* is greater than the cutoff value *C*, renal flare is predicted using model 15, where:


\begin{eqnarray*}Y &=& 1.16 \times \left( {{\mathrm{low}}\,{\mathrm{C}}3} \right) + 1.84 \times \left( {{\mathrm{positive}}\,{\mathrm{anti - dsDNA}}} \right) + 1.49 \nonumber\\
&&\times \left( {{\mathrm{presence}}\,{\mathrm{of}}\,{\mathrm{cellular}}/{\mathrm{fibrocellular}}\,{\mathrm{crescent}}} \right) + 2.21\nonumber\\
&&\times \left( {{\mathrm{presence}}\,{\mathrm{of}}\,{\mathrm{endocapillary}}\,{\mathrm{hypercellularity}}} \right) - 3.54\end{eqnarray*}

**Table 3:** A multivariate analysis of predicting factors for future LN flare within 3 years of maintenance therapy.

Variables^b^	OR	95%CI	*P* value
Proteinuria^a^	2.521	0.517–12.300	.253
Low C3	3.198	0.920–11.116	.067
Low C4	0.929	0.175–4.931	.931
Anti-dsDNA-positive	6.315	1.762–22.637	.005
Cellular/fibrocellular crescent	4.426	1.472–13.304	.008
Endocapillary hypercellularity	9.085	2.794–29.543	<.001
Fibrinoid necrosis/karyorrhexis	1.788	0.303–10.545	.521
Subendothelial deposits	1.707	0.482–6.048	.408
Acute tubular injury	0.782	0.173–3.544	.750

a Proteinuria was log-transformed.

b specific clinical variables and specific histologic variables at biopsy 2.

**Table 4:** logistic regression models to predict future LN flare.

Model	Predictor^a^,^c^	*P* value	Misclassification rate^b^	AUC
1	1	<.001	0.21	.75
2	2, 3, 4, 5	<.001	0.19	.76
3	3, 5	.001	0.19	.69
4	6	<.001	0.19	.85
5	7, 8, 9, 10, 11	<.001	0.15	.83
6	7, 8	<.001	0.18	.81
7	1, 6	<.001	0.17	.87
8	1, 7, 8, 9, 10, 11	<.001	0.15	.85
9	1, 7, 8	<.001	0.15	.85
10	2, 3, 4, 5, 6	<.001	0.19	.88
11	3, 5, 6	<.001	0.19	.88
12	2, 3, 4, 5, 7, 8, 9, 10, 11	<.001	0.16	.89
13	2, 3, 4, 5, 7, 8	<.001	0.16	.89
14	3, 5, 7, 8, 9, 10, 11	<.001	0.16	.89
15	3, 5, 7, 8	<.001	0.16	.89

a Predictors are defined as follows: 1 = SLEDAI-2 K, 2 = log (proteinuria), 3 = low C3, 4 = low C4, 5 = anti-dsDNA-positive, 6 = AI, 7 = cellular/fibrocellular crescent, 8 = endocapillary hypercellularity, 9 = fibrinoid necrosis/karyorrhexis, 10 = subendothelial deposits, 11 = acute tubular injury.

b Using a predicted probability of .5 as the cutoff.

c Variables at biopsy 2.

A *C* value of 0.18 gave the maximum sum of sensitivity (85.7%) and specificity (74.4%) in discriminating between flare and no flare.

The sensitivity analysis was performed on the seven patients excluded based on flare and no flare, and the conclusions obtained were consistent with these results.

## DISCUSSION

In this study, we found that histologic activity index after induction therapy was significantly better than the clinical response in predicting LN flare, and the effectiveness of the prediction model for LN flare was significantly improved after adding histologic variables into clinical variables. Patients with clinical remission who had residual active histologic lesions still had a high rate of LN flare, especially in the presence of cellular/fibrocellular crescent and endocapillary hypercellularity. These data suggest that examination of kidney histology after clinical remission of induction therapy may provide information that can be used to predict who is likely to flare and who is likely to remain in remission during maintenance treatment.

LN flare could not be identified by demographic, clinical and histologic variables before induction treatment. Previous studies have also shown that histologic variables before induction treatment were not predictors for relapse [17, 18], although Askenazi et al. [17] reported that children with proliferative LN who had proteinuria >2 g/d and estimated glomerular filtration rate (eGFR) <75 ml/min/1.73 m^2^ before induction therapy treated with cyclophosphamide were more likely to relapse.

The flare rate for patients with complete clinical remission after induction therapy was lower than those with partial clinical remission. However, no measurement at biopsy 2 used to evaluate the clinical remission, such as proteinuria, hematuria, and serum creatinine, was significantly associated with renal flare. Meanwhile, positive anti-dsDNA reflecting SLE activity were retained in the stepwise regression analysis, and low C3 increased the AUC of the prediction model and reduced misclassification rate.

Our study found that patients with AI > 1 at biopsy 2 had a higher risk of LN flare during maintenance treatment. Arends et al. [4] reported that a decrease in AI between two biopsies for proliferative LN after 2 years of immunosuppressant was the only independent inversely related predictor for LN flare. The LuFla study [19] found that patients who had biopsy in complete clinical remission for at least 12 months with an AI of 1 or more had a high risk of future LN flare, and everyone with an AI of >2 experienced flare. A cohort study demonstrated withdrawal of immunosuppression in patients with clinical remission and an AI of 0 at repeat biopsy reduced subsequent renal flares [20]. A low AI can identify patients with clinical remission for whom immunosuppressant withdrawal is safe [21] and relapse-free survival rate is high during maintenance therapy [22]. These findings indicate that histologic combined with clinical response provides a better chance of avoiding LN flares [23].

Only a limited number of active histologic lesions had prognostic value. The presence of cellular/fibrocellular crescent and endocapillary hypercellularity after induction therapy were found to be independent risk factors for LN flare. Hill et al. [24] found the composite variables of histologic inflammatory after induction therapy was a risk factor for LN relapse. A repeat biopsy study characterized active histologic lesions declined with successful treatment, but this took a considerable amount of time and the rate of resolution of individual histologic lesions was variable [25]. Endocapillary hypercellularity was one of the dominant ongoing active lesions in the individuals treated with >40 months [25]. The endocapillary hypercellularity also predicted flare better than did the combined AI in LuFla study [19]. These findings suggest that more weight should be given to specific active histologic lesions, especially extracapillary lesions.

There were no statistically significant differences in the induction and maintenance treatment regimens between the flare and no-flare groups. Most of the patients in this study were from previous multicenter clinical trials, treated and followed as protocol recommended [14, 15, 26–28]. We reviewed every record of the enrolled patients, and no treatment plan was made based on the results of biopsy 2, thus mitigating bias, which might be important for analyzing the predictive value of histologic variables at biopsy 2 on future LN flare. However, as a retrospective study, the most important limitation was that this study did not demonstrate that extension of induction therapy in patients with high histologic activity and clinical remission would have prevented future renal flares. This is a critical question and will need to be examined in a well-designed prospective study.

In conclusion, renal flare in LN patients with clinical remission is strongly associated with the residual active histologic lesions. Our results highlight the usefulness of kidney biopsy in evaluating disease status in patients with clinical remission, and the importance of pursue the histologic remission in the management of LN.

## Supplementary Material

sfae350_Supplemental_File

## Data Availability

The data underlying this article will be shared on reasonable request to the corresponding author.
