# Supplementary material for: Predictive value of residual active histologic lesions on renal flare in lupus nephritis patients with clinical remission
Source: Clin Kidney J. 2024 Nov 18;17(12):sfae350. doi: 10.1093/ckj/sfae350 (PMC11650015; doi:10.1093/ckj/sfae350)
Supplement: sfae350_Supplemental_File [file sfae350_supplemental_file.docx]

**Predictive value of residual active histologic lesions on renal flare in lupus nephritis patients with clinical remission**

**Supplementary material**

**Supplementary Table S1:** Comparison of induction and maintenance treatment regimens between the flare and no-flare groups.

**Supplementary Table S2:** Univariate logistic regression analysis of potential predictors for future LN flare within 3 years of maintenance therapy.

**Supplementary Figure S1:** Activity index of patients at biopsy 2 after induction therapy who experienced LN ﬂare and patients who did not during maintenance therapy within a 3-year follow-up period.

| **Supplementary Table S1 Comparison of induction and maintenance treatment regimens between the flare and no-flare groups** | | | | |
| --- | --- | --- | --- | --- |
| **Variable** | **Total (n=114)** | **Fare group (n=28)** | **No ﬂare group (n=86)** | ***P*** **value** |
| Induction treatment |  |  |  | 0.115 |
| P+CYC, n (%) | 19 (16.7) | 4 (14.3) | 15 (17.4) |  |
| P+MMF+FK506, n (%) | 57 (50.0) | 10 (35.7) | 47 (54.7) |  |
| P+MMF, n (%) | 15 (13.2) | 7 (25.0) | 8 (9.3) |  |
| P+FK506, n (%) | 23 (20.2) | 7 (25.0) | 16 (18.6) |  |
| Maintenance treatment |  |  |  | 0.346 |
| P+TW, n (%) | 16 (14.0) | 3 (10.7) | 13 (15.1) |  |
| P+MMF+FK506, n (%) | 20 (17.5) | 6 (21.4) | 14 (16.3) |  |
| P+MMF, n (%) | 24 (21.1) | 9 (32.1) | 15 (17.4) |  |
| P+FK506, n (%) | 5 (4.4) | 0 | 5 (5.8) |  |
| P+AZA, n (%) | 32 (28.1) | 5 (17.9) | 27 (31.4) |  |
| P+LFM, n (%) | 17 (14.9) | 5 (17.9) | 12 (14.0) |  |
| Note: P, prednisone; CYC, cyclophosphamide; MMF, mycophenolate mofetil; FK506, Tacrolimus; AZA, azathioprine; LFM, leflunomide; TW, tripterygium wilfordii glycosides. | | | | |

| **Supplementary Table S2 Univariate logistic regression analysis of potential predictors for future LN flare within 3 years of maintenance therapy** | | | |
| --- | --- | --- | --- |
|  | **OR** | **95%CI** | ***P*** **value** |
| Clinical predictors at biopsy 2 |  |  |  |
| SLEDAI-2K | 1.280 | 1.126, 1.454 | < 0.001 |
| Proteinuria ^a^ | 4.466 | 1.348, 14.789 | 0.014 |
| Low C3 | 3.990 | 1.508, 10.560 | 0.005 |
| Low C4 | 3.600 | 1.283, 10.098 | 0.015 |
| Anti-dsDNA-positive | 4.102 | 1.621, 10.380 | 0.003 |
| Histologic predictors at biopsy 2 |  |  |  |
| Activity index | 2.222 | 1.616, 3.056 | < 0.001 |
| Cellular/Fibrocellular crescent | 6.347 | 2.515, 16.020 | < 0.001 |
| Endocapillary hypercellularity | 8.569 | 3.300, 22.251 | < 0.001 |
| Fibrinoid necrosis/Karyorrhexis | 6.480 | 1.913, 21.945 | 0.003 |
| Subendothelial deposits | 3.633 | 1.390, 9.498 | 0.008 |
| Acute tubular injury | 3.422 | 1.172, 9.996 | 0.024 |
| ^a^ Proteinuria was log-transformed. | | | |


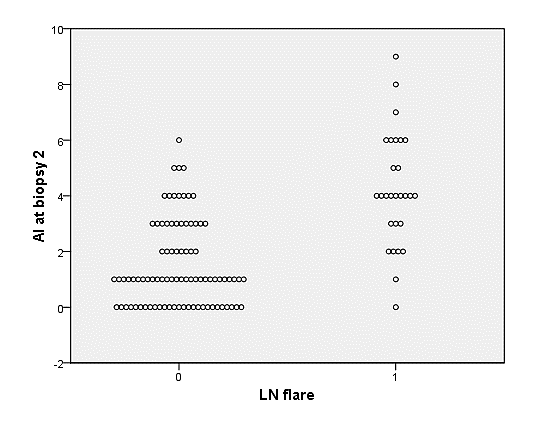


**Supplementary Figure S1: Activity index of patients at biopsy 2 after induction therapy who experienced LN ﬂare and patients who did not during maintenance therapy within a 3-year follow-up period**

Note: Each dot represents one patient. In the horizontal axis, 0 represents the no flare group, and 1 represents the flare group.

AI: Activity index; LN: Lupus nephritis.
